# Placental Vascular Pathology Associated with Congenital Lymphocytic Choriomeningitis Virus Infection, Philadelphia, Pennsylvania, USA

**DOI:** 10.3201/eid3206.260165

**Published:** 2026-06

**Authors:** Abin Abraham, Rebecca L. Linn, Dustin D. Flannery, Scott M. Gordon

**Affiliations:** Children’s Hospital of Philadelphia, Philadelphia, Pennsylvania, USA (A. Abraham, R.L. Linn, D.D. Flannery, S.M. Gordon); University of Pennsylvania Perelman School of Medicine, Philadelphia (R.L. Linn, D.D. Flannery, S.M. Gordon)

**Keywords:** Lymphocytic choriomeningitis virus, viruses, zoonoses, congenital viral infection, placenta, pregnancy, United States

## Abstract

Congenital lymphocytic choriomeningitis virus (LCMV) infection is associated with major neurologic malformations and fetal demise. We report 2 cases of probable congenital LCMV infection and chorioretinitis, cerebral ventriculomegaly, and placental histopathology in Philadelphia, Pennsylvania, USA. Clinicians who suspect congenital LCMV infection should screen for chorioretinitis, LCMV antibodies, and evidence of placental pathology.

Lymphocytic choriomeningitis virus (LCMV) is an underappreciated congenital pathogen carried by rodents (*1*). Congenital LCMV infection is associated with pathognomonic chorioretinitis and a variety of neurologic malformations (*2*). A case of fetal hydrops caused by congenital LCMV also has been reported (*3*). In a large maternal seroprevalence study, we found that 2.5% of a random sample of 1,000 women who gave birth in Philadelphia, Pennsylvania, USA, were LCMV IgG–positive (*4*). Those data support that pregnant women are at risk for LCMV exposure in a major urban area and that LCMV acquired during pregnancy can cause devastating short- and long-term outcomes for newborns.

The placenta nourishes the fetus in the womb and protects it from pathogens (*5*). Select pathogens can inflame and disrupt the structure and function of the placenta (*6*). The effects of congenital LCMV infection on the placenta remain unknown. Here, we provide evidence of inflammatory and vascular placental pathology in 2 cases of probable congenital LCMV infection in Philadelphia, diagnosed by clinical history, clinical findings, and neonatal and maternal serologic studies.

## The Cases

**Figure 1:**
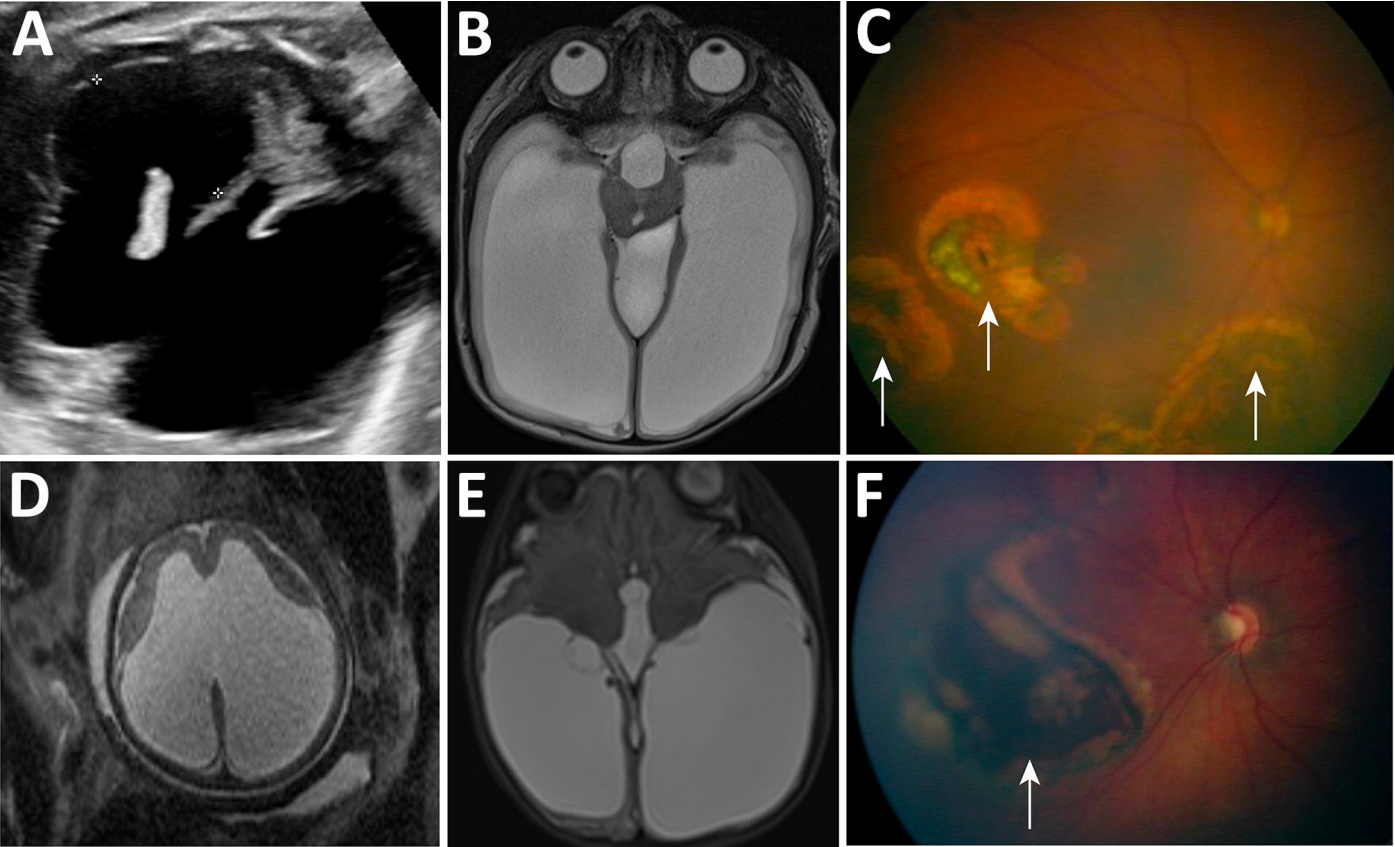
Imaging findings for ventriculomegaly and chorioretinitis associated with congenital lymphocytic choriomeningitis virus infection, Philadelphia, Pennsylvania, USA. A–C) Case 1; D–F) case 2. A) Routine ultrasonography at 36 weeks’ gestation, demonstrating severe cerebral ventriculomegaly in the fetus (black space between markers on the anatomic right and the unmarked black space on the anatomic left). B) Postnatal (38 weeks 4 days’ gestation) brain magnetic resonance imaging of newborn confirmed severe cerebral ventriculomegaly (large, white, fluid-filled spaces bilaterally and in the center of the image). C) Fundoscopic examination of newborn, revealing multiple areas of chorioretinitis (arrows). D) Routine ultrasonography at 36 weeks’ gestation, demonstrating severe cerebral ventriculomegaly in the fetus. E) Postnatal (38 weeks 5 days’ gestation) brain magnetic resonance imaging of newborn confirmed severe cerebral ventriculomegaly. F) Fundoscopic examination of newborn, revealing a large area of chorioretinitis (arrow).

The first case occurred in a 22-year-old G2P1 (2 pregnancies, 1 delivery) woman examined for multidisciplinary evaluation after severe fetal cerebral ventriculomegaly was identified on routine ultrasound growth scan at 36 weeks’ gestation (Figure 1, panel A). Umbilical artery, middle cerebral artery, and ductus venosus Doppler indices were within normal limits for gestational age. The anatomy scan at 26 weeks’ gestation revealed echogenic bowel but no other abnormalities. The pregnancy was complicated by gestational diabetes. Maternal prenatal laboratory testing demonstrated immunity to rubella virus, as well as prior exposure (IgG-positive) to cytomegalovirus (CMV) and *Toxoplasma*. The woman reported 2 pet dogs at home but denied exposure to rodents during pregnancy. She recalled subjective fever and chills at 16 weeks’ gestation.

A male infant was born by repeat cesarean delivery at 38 weeks 4 days’ gestation. Birthweight was at the 71st percentile and head circumference at the 97th percentile for age. Postnatal magnetic resonance imaging (MRI) confirmed cerebral ventriculomegaly, and ophthalmologic evaluation demonstrated chorioretinitis (Figure 1, panels B, C), prompting evaluation for congenital infection. Newborn laboratory evaluation showed *Toxoplasma* IgG-positive and IgM-negative and rubella virus IgM-negative on serology; PCR of urine was CMV-negative, and PCR of serum was herpes simplex virus (HSV)–negative. The infant’s LCMV IgG titer was 1:2,560 and LCMV IgM was negative (<1:10). Maternal LCMV serologic testing was not performed.

**Figure 2:**
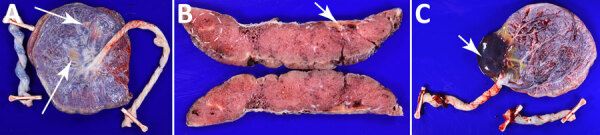
Pathologic findings of placental vascular anomalies associated with congenital lymphocytic choriomeningitis virus infection, Philadelphia, Pennsylvania, USA. A, B) Placenta from case 1; C) placenta from case 2. A) Gross pathology of the fetal surface showing yellow-white subchorionic intervillous thrombi (arrows). B) Cross sections of the placenta showing a subchorionic intervillous thrombus (arrow) and a peripheral intraparenchymal thrombus (white dashed circle). C) Gross pathology of the fetal surface of placenta with marginal insertion of three vessel umbilical cord and acute subamniotic hemorrhage (arrow). No additional gross lesions were identified after serial sectioning.

**Figure 3:**
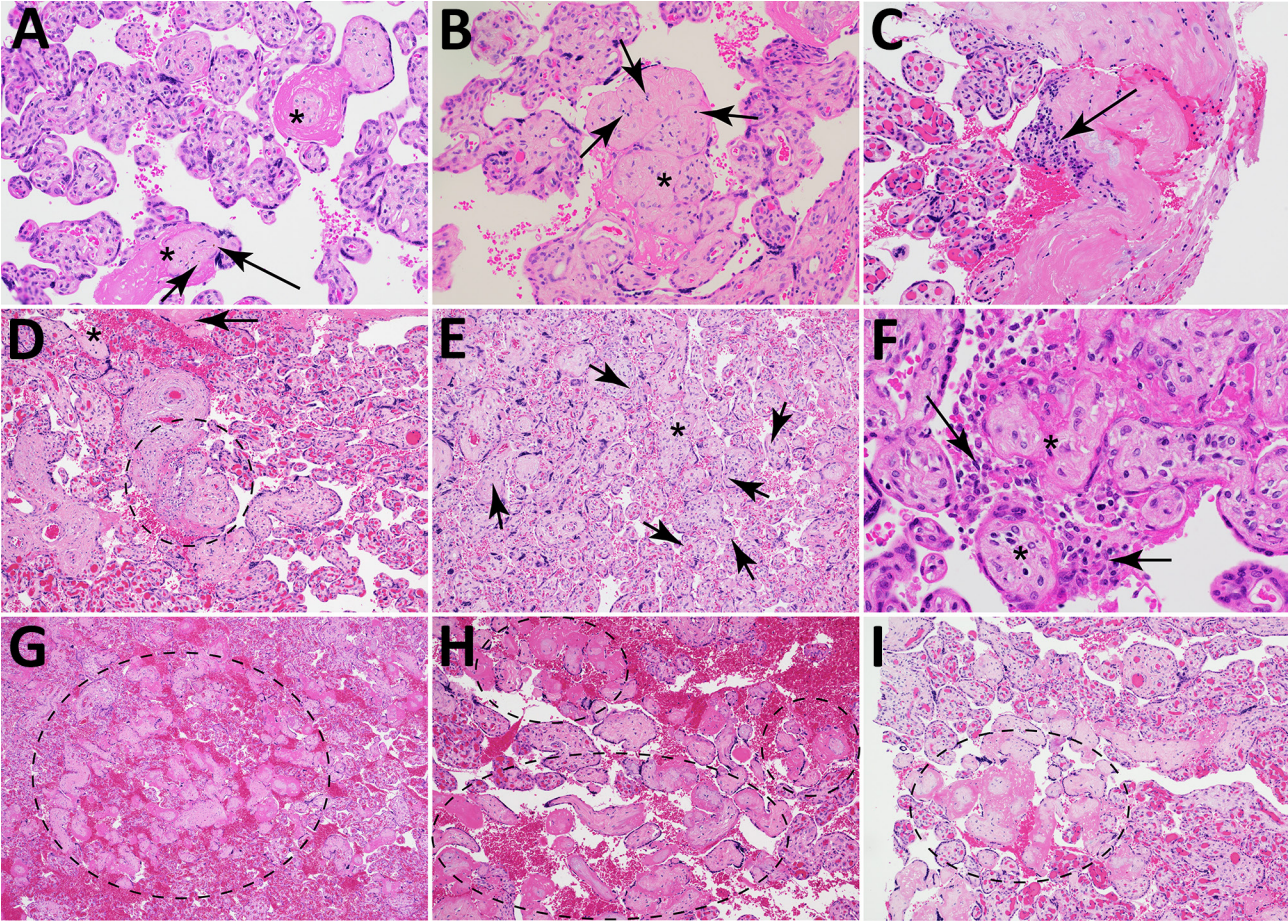
Microscopic findings for placental vascular pathology associated with congenital lymphocytic choriomeningitis virus infection, Philadelphia, Pennsylvania, USA. Hematoxylin and eosin–stained cross-sections of placenta from case 1 (A, B) and case 2 (C–I). A) Avascular villi surrounded by pink perivillous fibrin (asterisks) with scattered foci of rust-colored hemosiderin deposition (arrows) within the hyalinized stroma. Original magnification ×200. B) Cluster of avascular villi in the center of the image (asterisk) with scattered intravillous lymphocytes (arrows), compatible with chronic villitis. Original magnification ×200. C) Plasma cells (arrow) within the basal plate, consistent with chronic deciduitis. Original magnification ×200. D) Chronic villitis and perivillitis involving stem villi vessels (circle), with an associated avascular villus (asterisk) and fibrin deposition (arrow). Original magnification ×100. E) Chronic villitis (asterisk) with villous stromal vascular karyorrhexis; arrows point to examples of extravasated, fragmented erythrocytes and nuclear debris. Original magnification ×100. F) Histiocytic intervillositis (arrows) adjacent to avascular villi (asterisks) with perivillous fibrin deposition. Original magnification ×400. G–I) Avascular villi with associated perivillous fibrin deposition (dashed ellipses). G) Original magnification ×40; H, I) original magnification ×100.

Placental weight was at the 50th percentile for gestational age. Gross pathology showed 2 subchorionic intervillous thrombi and a peripheral intraparenchymal thrombus (Figure 2, panels A, B). Pathologic examination demonstrated high-grade fetal vascular malperfusion. We noted multiple foci of avascular villi, similar to that reported in congenital CMV infection (*7*), and some villi had stromal hemosiderin deposition (Figure 3, panels A, B). The maternal-fetal interface showed chronic deciduitis with plasma cell infiltration (not shown) and focal, low-grade chronic villitis in the placental parenchyma (Figure 3, panel B). We noted evidence of low-grade maternal vascular perfusion, and mural hypertrophy of membrane arterioles, mild accelerated villous maturation with patchy increase in syncytial knots (*8*,*9*), mildly increased perivillous fibrin deposition, and variable villous agglutination.

The second case occurred in a 27-year-old G5P4 woman examined for multidisciplinary evaluation at 38 weeks’ gestation. Routine ultrasound growth scan revealed severe fetal cerebral ventriculomegaly (Figure 1, panel D). Doppler velocimetry studies were within reference limits for gestational age. As in case 1, this mother had an unremarkable prenatal anatomy scan at 20 weeks’ gestation. Routine prenatal laboratory tests were unremarkable, and no additional testing for CMV, HSV, or *Toxoplasma* was performed. By maternal report, at 24 weeks’ gestation, she had severe meningitis diagnosed during a 2-week hospital admission. A causative agent was not identified, and the mother reported recovering fully from the illness. The pregnancy was otherwise uncomplicated.

A female infant was born at 38 weeks 5 days’ gestation by primary cesarean delivery because of fetal hydrocephalus and macrocephaly. Birthweight was at the 16th percentile and head circumference at the 54th percentile for age. Postnatal neuroimaging demonstrated severe ventriculomegaly, and postnatal ophthalmologic examination demonstrated chorioretinitis (Figure 1, panels E, F). Therefore, we evaluated the newborn for congenital infections; serum test results were negative for *Toxoplasmosis* IgM and IgG and rubella IgM, and PCR on urine was CMV-negative. The newborn’s serum LCMV IgG was 1:2,560; LCMV IgM was negative (<1:10).

Upon further questioning, the mother revealed exposure to a rodent infestation in the home during pregnancy. The mother’s serum LCMV IgG was 1:10,240 and was IgM-positive at 1:10. The maternal exposure to rodent excreta, plus meningitis, serologic data, and timing of the fetal ventriculomegaly, suggest a diagnosis of congenital LCMV infection acquired during the second trimester.

Placental weight was less than the 10th percentile for gestational age. Gross pathology showed an acute subamniotic hemorrhage (Figure 2, panel C). The placenta in case 2 exhibited inflammatory changes of the maternal–fetal interface, including mild chronic deciduitis with plasma cell infiltrates (Figure 3, panel C), chronic chorionitis (not shown), multifocal low-grade chronic villitis, and perivillitis involving terminal and stem villi (Figure 3, panels D, E). The placenta from case 2 also exhibited focal histiocytic intervillositis (Figure 3, panel F), defined by inappropriate accumulation of macrophages into the intervillous space and associated with fetal growth restriction or demise (*10*,*11*). We noted multiple foci of avascular villi and villous stromal vascular karyorrhexis, often associated with perivillous fibrin deposition (Figure 3, panels E–I). Those pathologic changes are compatible with high-grade fetal vascular malperfusion (*12*). Given the proximity of the microanatomic abnormalities and inflammation, the placental pathology might reflect progression of the inflammatory changes observed.

The clinical, radiographic, ophthalmologic, and serologic data in the 2 cases we describe support probable congenital LCMV infection, acquired during the 2nd trimester of pregnancy, with profound long-term consequences. Both children underwent ventriculoperitoneal shunt placement for hydrocephalus and endured several episodes of shunt malfunctions requiring shunt revisions. The child in case 1 had hemiplegia, obstructive sleep apnea, and motor and cognitive delays diagnosed. The child in case 2 had spastic quadriplegic cerebral palsy, epilepsy, and motor and cognitive delays diagnosed. Both children remain dependent on multiple subspecialists, an array of medical technologies, and aggressive physical, occupational, and speech therapy regimens. 

## Conclusions

Whole-exome sequencing was unrevealing for either case in this report. In an era of increasingly accessible but costly and time-intensive genetic testing, obstetric and neonatal providers should consider congenital LCMV in the differential diagnosis of congenital neurologic malformations. Definitive diagnosis of any congenital infection remains challenging, however. Diagnosis is often made retrospectively, long after acute maternal infection. Mothers can exhibit minimal systemic symptoms (as in case 1) or severe clinical illness (as in case 2). Because few readily available tools are available to identify LCMV within the placenta, clinicians have relied on a constellation of clinical, imaging, and serologic findings in the mother and infant to diagnose congenital LCMV infection (*13*). 

To establish causality between LCMV and congenital findings in the future, detecting LCMV within the placenta of suspected cases will be essential. In the meantime, we propose that infants with suspected congenital infection undergo ophthalmologic screening for chorioretinitis and serologic testing for LCMV antibodies. We also recommend maternal serologic testing to support the diagnosis and provide insight into the timing of infection. Preventing congenital LCMV requires minimizing prenatal exposures and halting vertical transmission during pregnancy. Future mechanistic investigations into LCMV-associated fetoplacental injury are urgently needed. Then, we can develop new therapies to preserve healthy placental function and normal fetal neurodevelopment in the setting of congenital LCMV.
